# *Pseudomonas aeruginosa* reverse diauxie is a multidimensional, optimized, resource utilization strategy

**DOI:** 10.1038/s41598-020-80522-8

**Published:** 2021-01-14

**Authors:** S. Lee McGill, Yeni Yung, Kristopher A. Hunt, Michael A. Henson, Luke Hanley, Ross P. Carlson

**Affiliations:** 1 Department of Chemical and Biological Engineering, Center for Biofilm Engineering, Montana State University, Bozeman, MT 59717 USA; 2 Department of Microbiology and Immunology, Montana State University, Bozeman, MT 59717 USA; 3 Department of Chemistry, University of Illinois at Chicago, Chicago, IL 60607 USA; 4 Department of Civil and Environmental Engineering, University of Washington, Seattle, WA 98115 USA; 5 Department of Chemical Engineering, Institute for Applied Life Sciences, University of Massachusetts, Amherst, MA 01003 USA

**Keywords:** Metabolomics, Proteomics, Biochemical reaction networks, Computational models, Bacteria, Microbial ecology

## Abstract

*Pseudomonas aeruginosa* is a globally-distributed bacterium often found in medical infections. The opportunistic pathogen uses a different, carbon catabolite repression (CCR) strategy than many, model microorganisms. It does not utilize a classic diauxie phenotype, nor does it follow common systems biology assumptions including preferential consumption of glucose with an ‘overflow’ metabolism. Despite these contradictions, *P. aeruginosa* is competitive in many, disparate environments underscoring knowledge gaps in microbial ecology and systems biology. Physiological, omics, and in silico analyses were used to quantify the *P. aeruginosa* CCR strategy known as ‘reverse diauxie’. An ecological basis of reverse diauxie was identified using a genome-scale, metabolic model interrogated with in vitro omics data. Reverse diauxie preference for lower energy, nonfermentable carbon sources, such as acetate or succinate over glucose, was predicted using a multidimensional strategy which minimized resource investment into central metabolism while completely oxidizing substrates. Application of a common, in silico optimization criterion, which maximizes growth rate, did not predict the reverse diauxie phenotypes. This study quantifies *P. aeruginosa* metabolic strategies foundational to its wide distribution and virulence including its potentially, mutualistic interactions with microorganisms found commonly in the environment and in medical infections.

## Introduction

*Pseudomonas aeruginosa* is an opportunistic pathogen commonly isolated from diabetic ulcers, burn wounds, and battlefield injuries, as well as from the lungs of patients with cystic fibrosis (CF)^1–3^. Its presence is correlated with high patient morbidity and mortality^4–6^. *P. aeruginosa* is found in ~ 80% of chronic, diabetic ulcers which cost the US medical system $20–50 billion per year to treat^4–6^. *P. aeruginosa* virulence and persistence mechanisms are enabled across strains by a pangenome possessing approximately 5,200 core genes^7^. Maintaining the large genome and implementing the myriad of virulence strategies necessitates effective strategies for nutrient acquisition and nutrient allocation to metabolic pathways. While foundational to its global distribution and virulence, the basis of the *P. aeruginosa* central metabolism is poorly understood^4,6^.

Global regulatory systems select preferred carbon sources from pools of substrates in a process known as carbon catabolite control (CCC) or carbon catabolite repression (CCR)^8^. The best studied examples of CCR are from *Escherichia coli* and *Bacillus subtilis*^8–10^*.* The metabolic designs of these model organisms, which prefer glucose over other substrates, form the basis of most textbook CCR examples^11^. The CCR strategy represented by *E. coli* and *B. subtilis* is referred to here as ‘classic carbon catabolite repression’ (cCCR) to distinguish it from the broader CCR term. *P. aeruginosa* does not display a cCCR phenotype. Instead, this competitive microorganism, as evidenced by its global distribution which is arguably broader than *E. coli*^12–14^, has substrate preferences that are almost opposite of *E. coli*. *P. aeruginosa* utilizes a CCR strategy termed ‘reverse diauxie’ or reverse CCR (rCCR) which is defined by a hierarchy of preferred carbon sources that is nearly reverse that of cCCR preferences^8,15^. *P. aeruginosa* can readily catabolize glucose although it is not a preferred substrate, instead this bacterium preferentially catabolizes less energetic, nonfermentable substrates like succinate. The contrarian hierarchy of preferred carbon sources is proposed to be central to the versatility of *P. aeruginosa*. The ecological basis of rCCR is an open question with few published theories^8,9,16,17^. A quantitative understanding of rCCR lags cCCR. This is a critical knowledge gap that contributes to degradation of patient quality of life and costs society tens of billions of dollars per year^5^.

Natural environments do not permit unconstrained microbial growth^18^. Instead, life is constrained by the availability of resources such as reduced carbon or nitrogen sources^18^. Phenotypic plasticity can permit microorganisms to acclimate to resource scarcity^19–21^. In silico systems biology approaches have investigated resource investments (e.g. carbon, nitrogen) into different metabolic pathways via the enzyme synthesis requirements. The in silico methodologies, often referred to as resource allocation analysis or metabolic tradeoff theory, are powerful tools for predicting and interpreting phenotypes and have been applied extensively to cCCR microorganisms *E. coli* and *B. subtilis*^19,22–30^. For example, in silico and in vitro studies of *E. coli* quantified acclimation to carbon, nitrogen, or iron limitation along a metabolic tradeoff surface by optimizing the functional return on the limiting nutrient, at the expense of substrates found in excess^21,31^. This strategy resulted in ‘overflow metabolisms’ with the secretion of byproducts like acetate and lactate; overflow metabolisms are also known as the Warburg or Crabtree effect in eukaryotes^32^. Resource allocation analysis has not been applied to rCCR organisms. Given the large genomic potential and phenotypic plasticity of *P. aeruginosa*, these approaches hold potential for decoding the metabolic organization of this problematic bacterium.

Here, the ecological basis of *P. aeruginosa* rCCR was tested using a combination of physiological studies, exometabolomics, proteomics, and systems biology. The preference for substrates was measured and phenotypic characteristics, like the general lack of an overflow metabolism, were quantified. Proteomics measured a constitutive core metabolism centered on respiration and a dynamic set of enzymatic pathways that catabolized specific substrates, directing intermediates toward the core metabolism. The experimental data was analyzed with a genome-scale, metabolic model of *P. aeruginosa* and flux balance analysis (FBA) to identify ecological theories that predicted the observed phenotypes. *P. aeruginosa* did not optimize substrate preference based on standard systems biology assumptions such as the maximization of growth rate, as is commonly applied to cCCR phenotypes. Instead, *P. aeruginosa* metabolism was organized around a multidimensional, resource utilization strategy with constitutive expression of a respiration-based, core metabolism and substrate preferences that were based on minimizing the nutrient investment required to completely oxidize the substrate. Understanding a molecular-level basis of substrate preference, energy metabolism, and cell growth is foundational to controlling virulence mechanisms in *P. aeruginosa* including consortial interactions.

## Results

### Growth physiology and substrate preference of rCCR

**Figure 1:**
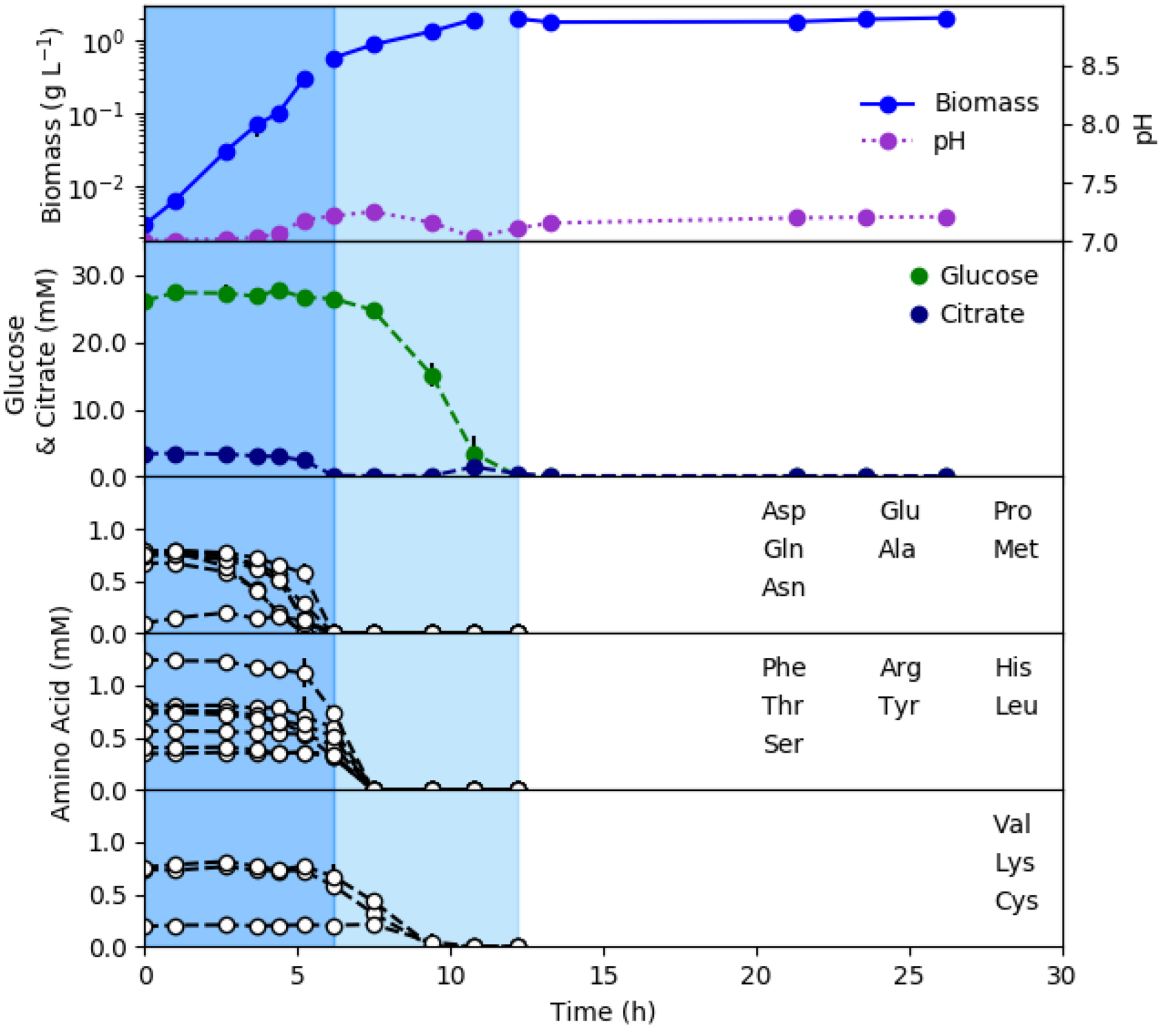
*P. aeruginosa 215* was grown in chemically-defined, glucose containing, medium (CSP G) in batch culture. Cultures demonstrated two exponential growth phases highlighted with different background shading. Amino acids were binned into three categories based on their time of exhaustion. Top tier amino acids were consumed during the first exponential growth phase while lower tier amino acids and glucose were consumed during the second exponential growth phase. All values are averages of three biological replicates, and metabolite values are also averaged from two technical replicates. Additional data can be found in supplementary material S3.

*Pseudomonas aeruginosa* strain 215 (Pa 215) is a medical isolate from a chronic wound^33,34^. Pa 215 was grown in chemically-defined, glucose containing, CSP G medium (materials and methods, supplementary material S1). Cultures exhibited two distinct exponential growth phases followed by stationary phase (Fig. 1). A subset of amino acids was consumed preferentially during the first exponential growth phase, which had the highest specific growth rate (Fig. 1, supplementary material S2). The second exponential growth phase corresponded with the catabolism of second and third tier amino acids and glucose. CSP G media contained a small concentration (3 mM) of citrate, which was added as an ion chelator; however, the citrate was readily catabolized as a preferred substrate during the first growth phase. The cultures did not exhibit an overflow metabolism defined by the secretion of reduced metabolic byproducts like acetate, as is typical of microorganisms expressing cCCR phenotypes^17,30^. Trace amounts of gluconate were secreted during glucose metabolism but were quickly depleted (supplementary material S3). Amino acid deamination products like α-ketoglutarate and pyruvate were not observed in spent medium or found in only trace amounts (< 1 mm), respectively.

**Figure 2:**
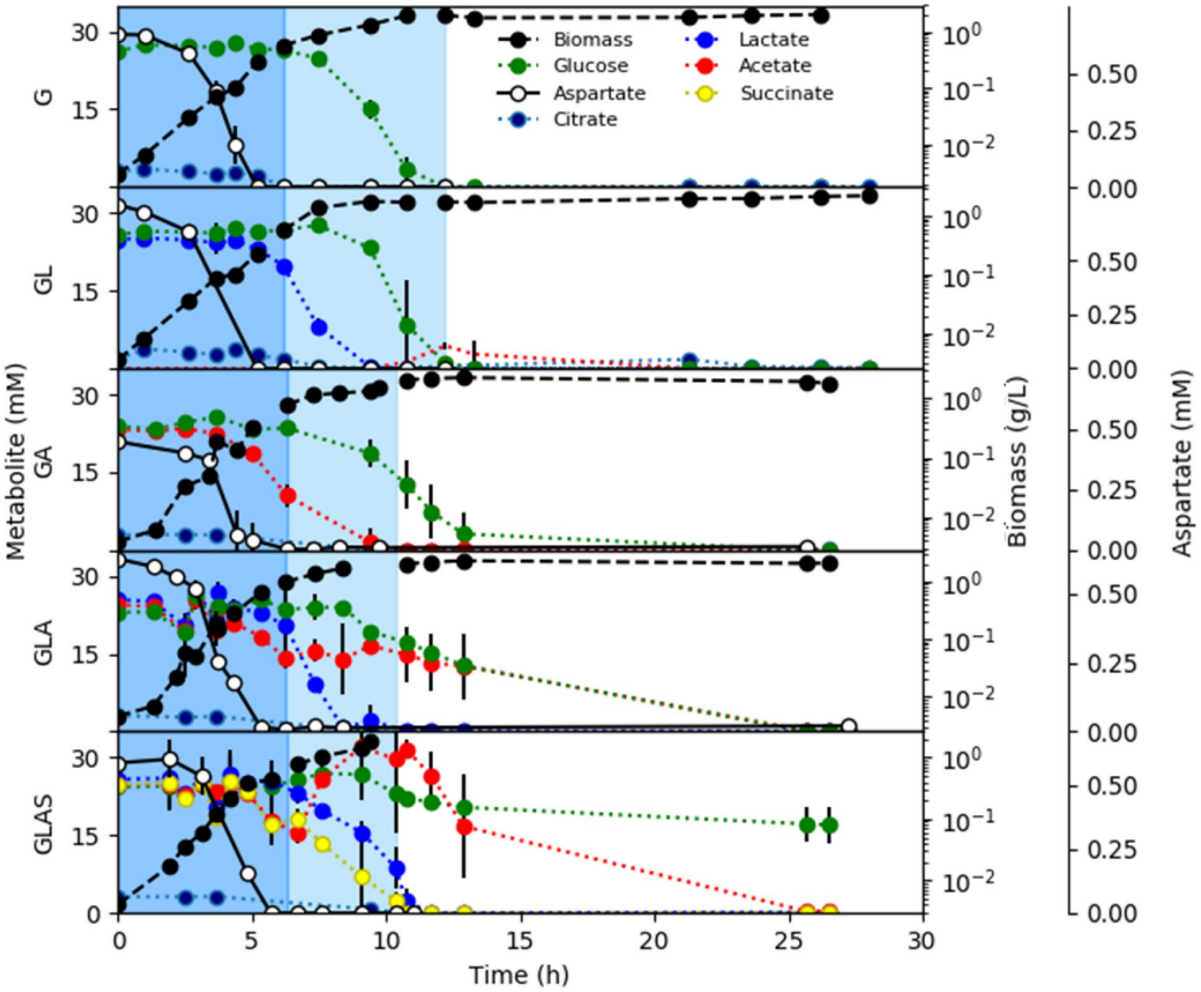
*P. aeruginosa* 215 substrate utilization order during batch growth in chemically-defined media. Panels plot biomass and substrate concentrations as a function of time for five medium configurations supplemented with different carbon sources at a concentration of 22 mM each. G = glucose, GL = glucose and lactate, GA = glucose and acetate, GLA = glucose, lactate, and acetate, GLAS = glucose, lactate, acetate, and succinate. Aspartate concentration (second y-axis) is plotted as a representative top tier amino acid. Cultures demonstrated two exponential growth phases highlighted with different background shading. Medium composition and additional data for each culture can be found in the supplementary material. All substrate values are averages of three biological replicates and two technical replicates.

Substrate utilization order for Pa 215 was quantified using five different formulations of CSP G medium supplemented with permutations of additional carbon sources: lactate (L), acetate (A), and succinate (S) (Fig. 2). Pa 215 grown on CSP GL medium, preferentially consumed the top tier amino acids, represented by aspartate in Fig. 2, followed by lower tier amino acids (data for each measured amino acid can be found in supplementary material S3–S8) and lactate before finally catabolizing glucose. Glucose catabolism was not observed while lactate was present. Pa 215 grown on CSP GA consumed the top tier amino acids followed by lower tier amino acids and acetate and finally glucose after the acetate was exhausted. Pa 215 grown on CSP GLA preferentially consumed top tier amino acids, then lower tier amino acids and lactate followed by acetate and glucose. Finally, Pa 215 grown on CSP GLAS preferentially catabolized the top tier amino acids, followed by succinate, lactate, acetate, and ultimately glucose. Ion chelator, citrate, was readily catabolized as a preferred substrate in all media formulations. No or minimal overflow metabolism (< 4 mM acetate, ~ 3% of lactate and glucose carbon moles in CSP GL medium) was observed. An exception was CSP GLAS grown cultures which accumulated acetate (~ 10 mM) above the initial medium concentrations. Upon exhaustion of succinate and lactate, the acetate was catabolized prior to glucose catabolism. The glucose was not completely catabolized in CSP GLAS medium because the medium was nitrogen limited (supplementary material S1). Culture parameters are summarized in supplementary material S2 and data is available in supplementary material S3–S7.

The order of amino acid catabolism was assessed for all five CSP medium formulations by binning the amino acids into three categories based on their time of exhaustion (Table 1). Binning was used, as opposed to using an absolute time metric, because each medium formulation had a different number of substrates leading to different total growth times. Other substrate usage metrics were considered including the initial time of substrate catabolism and nonlinear fitting of the temporal metabolite profiles to calculate a substrate ‘half-life’ value^35^; these metrics were sensitive to experimental variability during the initial growth phase where small fluctuations in substrate concentration, based likely on analytical techniques, influenced predictions. All amino acid data, including bin designations, time metrics, and fitting parameters, can be found in supplementary material S8. The preferred amino acids, referred to here as top tier, included aspartate, asparagine, glutamine, glutamate, and alanine. Most top tier amino acids were binned consistently across the five CSP media formulations as quantitated by their small standard deviations. The middle tier amino acids had more variability which may have been CCR-related or based on the temporal granularity of the experimental sampling schedule. The temporal trends in medium pH reflected the metabolism of different substrates. Catabolism of amino acids increased medium pH based on nitrogen chemistry. Catabolism of organic acids also raised the culture pH because the bacterium imports the protonated base, removing a proton from the medium.

**Table 1:** Amino acid utilization order for *P. aeruginosa* 215 cultures grown on five different, chemically-defined media supplemented with various additional carbon sources.

AA	Score
L-Asn	1 ± 0
L-Asp	1 ± 0
L-Glu	1 ± 0
L-Ala	1.2 ± 0.45
L-Pro	1.4 ± 0.89
L-Gln	1.5 ± 1
Gly	1.8 ± 0.45
L-Thr	1.8 ± 0.45
L-Arg	2 ± 0
L-Iso	2 ± 0.71
L-Ser	2 ± 0
L-Leu	2.2 ± 0.45
L-Val	2.8 ± 0.45
L-Lys	3 ± 0

Amino acids were binned into three categories (1, 2, 3) based on the time of exhaustion, averaged between three biological replicates for each of five medium conditions, n = 15. Data can be found in supplementary material S8.

Approximately 90% of the anabolic nitrogen in CSP G medium was in the form of amino acids (supplementary data S1). *P. aeruginosa* can use ammonium as the sole nitrogen source^36^. CSP G medium formulations were modified with the addition of 2 g/L ammonium chloride to test the effect of nitrogen form. The rCCR phenotype was not changed by the presence of ammonium. The cultures consumed the amino acids as preferred substrates followed by lactate and then glucose (supplementary material S9). The amino acid utilization order remained largely unchanged (supplementary material S10).

The common laboratory strain of *P. aeruginosa*, PAO1, was also grown on CSP GLAS medium. The PAO1 substrate utilization order of organic acids and glucose was the same as Pa 215 and the order of amino acid consumption was very similar to Pa 215 (supplementary material S11).

### Proteomics quantifies a constitutive, respiration-centric metabolism

Proteomic data were collected from CSP G and CSP GL grown cultures. Proteomic data are more predictive of cell function than transcriptomic or genomic data alone because they represent an actual allocation of resources into relatively stable, macromolecular pools^37,38^. Phenotypes were analyzed using label-free proteomics with mass-spectrometry (MS) of whole-cell lysates collected mid-first, exponential growth phase (4 h), early-second, exponential growth phase (7 h), and late-second, exponential growth phase (11 h). The proteomics data were analyzed with focus on central metabolism proteins associated with catabolizing the available substrates and with producing cellular energy.

**Figure 3:**
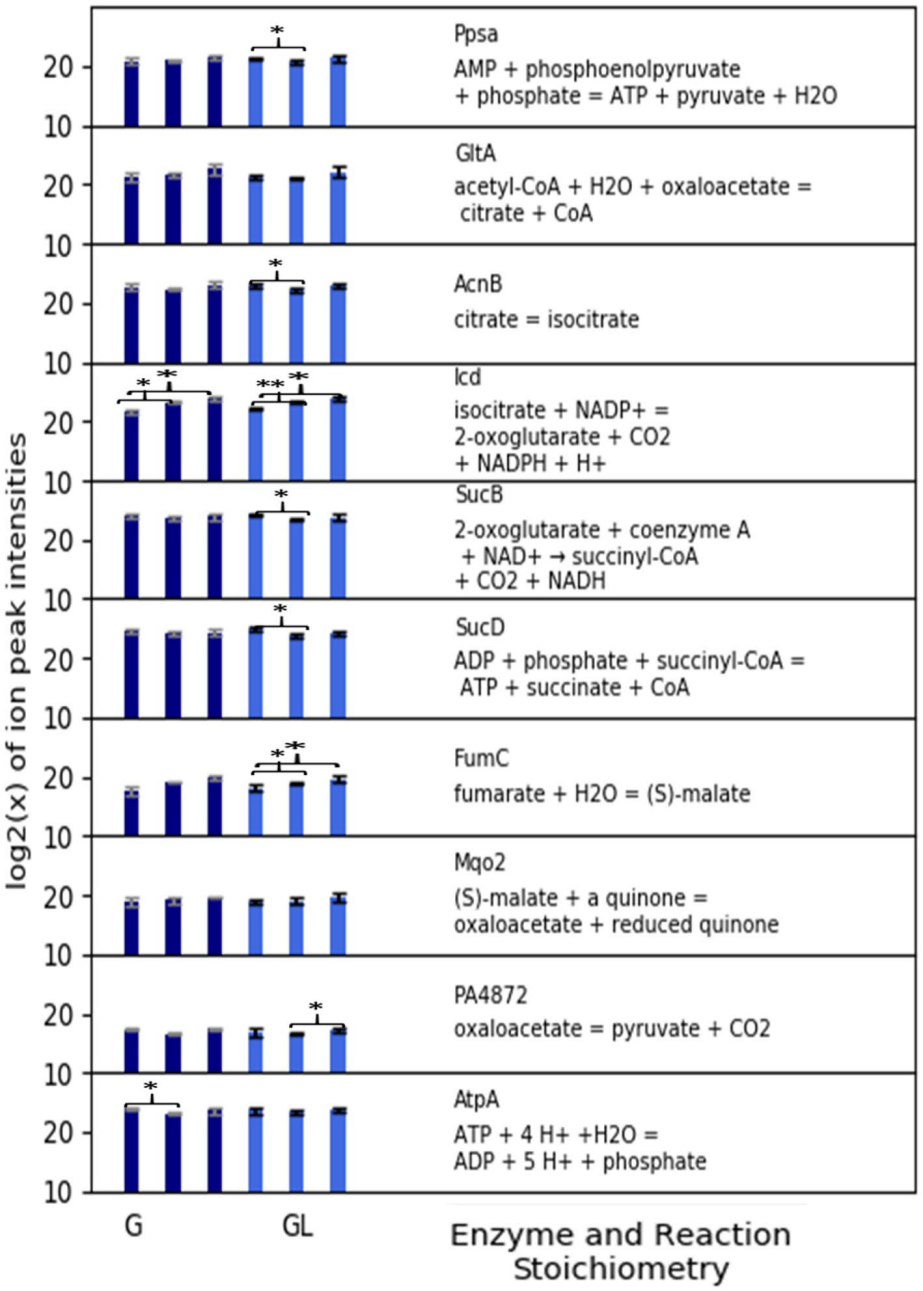
Proteomics data for *P. aeruginosa* 215 grown on chemically-defined, CSP G and CSP GL medium which contained glucose (G) or glucose and lactate (GL), respectively. CSP G culture data are represented by dark blue bars and CSP GL culture data are represented by light blue bars. Bars quantify the abundance of the enzyme during the first exponential growth phase (4 h), early second exponential growth phase (7 h), and late second exponential growth phase (11 h). Presented enzymes are involved in the tricarboxylic acid (TCA) cycle, anaplerotic reactions, and ATP synthesis. All values are averaged from three biological replicates. *p value < 0.05; **p value < 0.01.

Enzymes from the tricarboxylic acid (TCA) cycle and associated auxiliary enzymes had largely, constitutive abundances regardless of the medium formulation and the growth phase (Fig. 3). All TCA cycle enzymes except the membrane-associated succinate dehydrogenase were detected and quantified. Additionally, the enzymes oxaloacetate decarboxylase (PA4872) and PEP synthase which process metabolic intermediates from the TCA cycle for gluconeogenesis, were expressed constitutively. The abundance of ATP synthase subunits was also constitutive. Membrane-associated, electron transport chain (ETC) enzymes were not detected. It was assumed that the ETC enzymes were also constitutively expressed based on the TCA cycle and the ATP synthase protein abundances and the lack of an overflow metabolism.

**Figure 4:**
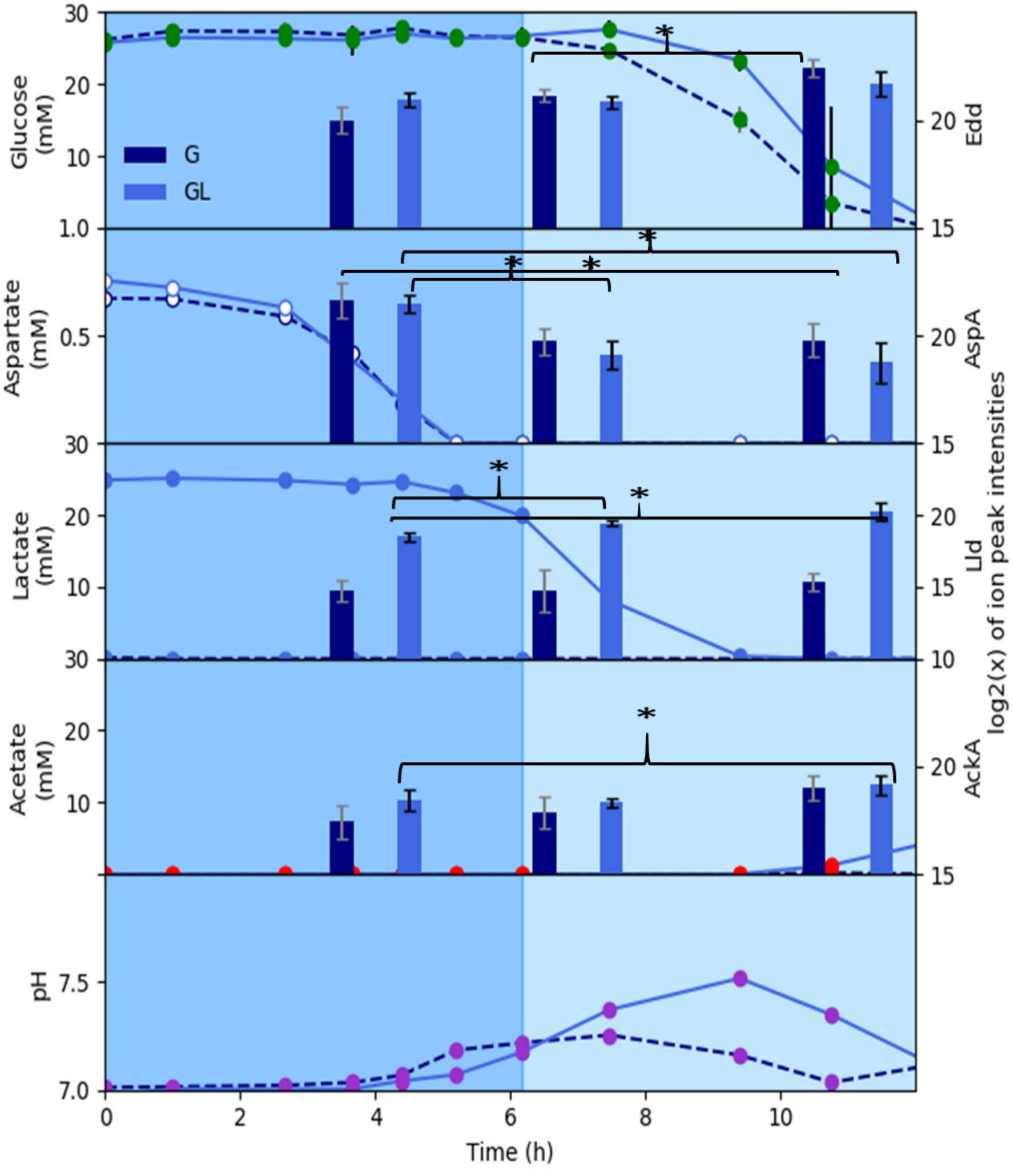
Substrate-specific, protein abundances for *P. aeruginosa* 215 cultures grown on chemically-defined CSP G (dark blue bars and dashed lines) and CSP GL (light blue bars and solid lines) media which contain glucose and glucose + lactate, respectively. Substrate concentrations for each enzyme are plotted in the same panel to highlight relationships. The three bars represent batch growth time points 4, 7, and 11 h. Metabolite values are averaged from three biological replicates and two technical replicates. Protein values are averaged from three biological replicates. *p value < 0.05; **p value < 0.01.

Enzymes associated with the processing of specific substrates did change in abundance based on presence and concentration of substrates, contrary to most TCA cycle enzymes (Fig. 4). Aspartate was plotted as a representative top tier amino acid (Table 1). Protein abundance for aspartate ammonia-lyse (AspA), responsible for the catabolism of aspartate, was elevated during the first exponential growth phase. When aspartate was exhausted, the abundance of AspA dropped as the metabolism shifted to other substrates. Following the depletion of top tier amino acids, the presence or absence of lactate was correlated with increasing or minimal abundances of lactate dehydrogenase protein (Lld), respectively. Pa 215 catabolized glucose, like all Pseudomonads, via the Enter-Doudoroff (ED) pathway^36,39^. Abundance of ED phosphogluconate dehydratase (Edd) increased while glucose was being metabolized, after the exhaustion of top tier amino acids. Acetate kinase (AckA) abundance increased for the CSP GL culture at the exhaustion of lactate and glucose. AckA quickly metabolized the small amount of acetate (< 4 mM) secreted at the exhaustion of lactate and glucose.

Proteomic analysis measured additional proteins that displayed changes in expression during exponential growth and stationary phases. Data can be found at ftp://massive.ucsd.edu/MSV000085590/.

### In silico analysis of rCCR phenotypes

CCR is a regulation scheme that contributes to metabolic plasticity. CCR regulation schemes have evolved to control expression of metabolic strategies that favor cellular fitness. The order of substrate utilization is hypothesized to reflect the ecological strategy used by *P. aeruginosa* to thrive in environmental and medical niches. Computational systems biology was used to test hypotheses regarding what fitness properties were being optimized in the Pa 215 cultures, with predictions compared to experimental data. In silico analyses used flux balance analysis (FBA) of a published, genome-scale, metabolic model of *P. aeruginosa* updated here with genome-supported, amino acid catabolism reactions^40,41^ (supplementary material S12). Stoichiometric modeling methods, such as FBA, can be utilized with a minimum number of a priori fitting parameters. The applied FBA considered only steady state simulations. Temporally-resolved simulations require enzyme kinetic parameters for every considered substrate, which are not available in the literature for *P. aeruginosa*.

**Figure 5:**
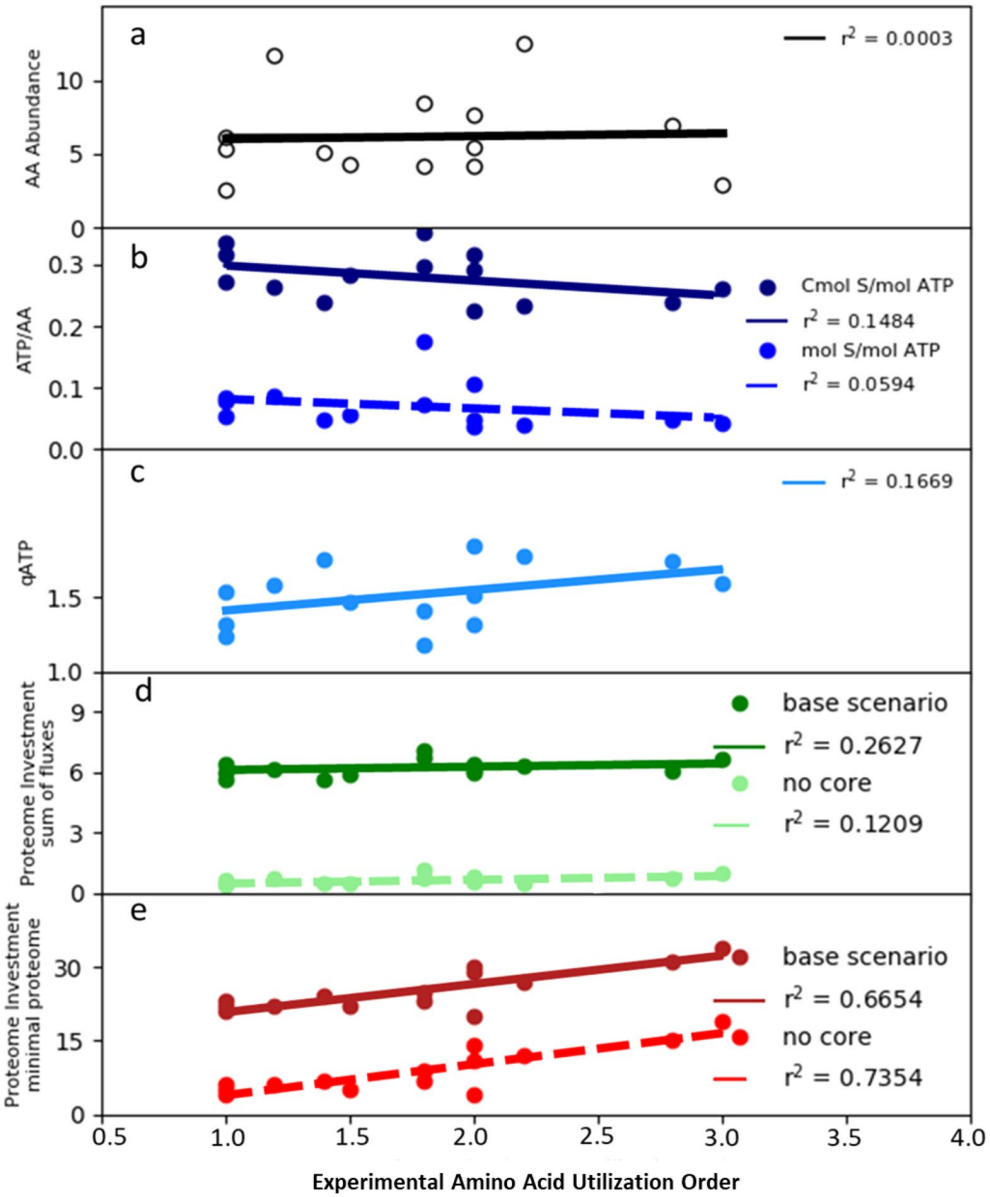
In silico analysis of reverse carbon catabolite repression (rCCR) substrate utilization order in *P. aeruginosa* 215. (**a**) Amino acid frequency in genome open reading frames plotted as a function of the experimental amino acid utilization order. (**b**) Optimal in silico cellular energy yield from the catabolism of each amino acid plotted as a function of experimental amino acid utilization order. Analysis considered both moles of amino acid and carbon moles (Cmol) of amino acid. (**c**) in silico maximization of cellular energy rate (qATP = mmol ATP g cdw^−1^ h^−1^) plotted as a function of experimental amino acid utilization order. Enzyme parameters were based on a survey study of catabolic enzymes (0.5 mmol AA g cdw^−1^ h^−1^, K_m_ = 0.2 mM, and CSP G medium composition, see supplementary material S1). (**d**) Computational analysis of amino acid utilization order based on resource investment into proteome for complete oxidation of substrate using the sum of fluxes as a proxy for proteome investment. Analysis considered a base metabolism scenario where all enzyme-catalyzed reactions were considered and a refined, no core metabolism scenario where only enzyme-catalyzed reactions extraneous to the experimentally-measured, constitutive proteome core where considered. A subset of the core enzymes is shown in Fig. 3 while an explicit list can be found in supplementary material S15. (**e**) Proteome investment analysis using the minimal proteome investment proxy to predict experimental amino acid utilization order. Analysis considered base metabolism scenario and refined, no core metabolism scenario.

In silico testing of ecological strategies was applied first to amino acid utilization order and included all experimentally measured amino acids except for aromatic and sulfur containing amino acids due to their specialty chemistries. The experimental amino acid utilization order did not correlate with the amino acid frequency in genome open reading frames (Fig. 5a) indicating the amino acids were not consumed solely for protein assembly; amino acids were also used as anabolic building blocks for other macromolecules and catabolized for cellular energy (supplementary material S8). Therefore, simulations considered either the production of cellular energy (e.g*.* ATP) or cellular growth which was quantified as carbon moles (Cmol) of biomass.

The first round of in silico analyses considered six separate, single dimension, optimization criteria which were informed by previous studies that examined numerous optimization criteria^22,30,42^. The criteria included (1) maximizing biomass or energy production rates based on electron donor, (2) maximizing biomass or energy production rates based on electron acceptor (O_2_), or (3) minimizing nutrient investment into the proteome required for either biomass or energy synthesis. The results of these simulations are presented in the next two sections and a summary of the results and analyses can be found in supplementary material S13.

### Amino acid utilization order did not correlate with in silico maximization of rates

Computational approaches for studying metabolism often assume cells utilize metabolic potential to maximize growth rate^43–45^. The experimental amino acid utilization order, as quantified across five media formulations (Table 1), was analyzed using this maximum rate theory. Separate, steady state simulations were run for each individual amino acid and for either biomass production or cellular energy production. Simulations identified the optimal phenotypes for the conversion of each substrate into product. The in silico phenotypes which maximized electron donor yields also mineralized the substrates secreting only CO_2_, consistent with in vitro cultures which did not utilize overflow metabolisms.

Maximizing cellular energy yield per amino acid, on a mole substrate or Cmol substrate basis, did not correlate with amino acid utilization order, having r^2^ values of 0.06 and 0.15 respectively (Fig. 5b). The in silico phenotypes which maximized yields were used to calculate maximum product rates using enzyme parameters from survey studies^46,47^ and experimental medium composition (supplementary material S1). This common theoretical treatment linked product yields to product rates such that maximizing one maximizes the other, this was an assumption of convenience and the advantages and disadvantages of its application have been discussed in the literature^30,43,48^. Maximizing the rate of cellular energy production (or growth) did not predict amino acid utilization order, as the correlation was r^2^ = 0.17 (Fig. 5c) (supplementary material S13–S18).

*P. aeruginosa* has a respiration-centric metabolism. The rate maximization criterion was also applied to O_2_ which is required to mineralize the amino acids under the experimental conditions. This alternative, rate maximization criterion predicted substantial overflow metabolisms for most of the amino acids. This predicted phenotypic trait was not consistent with the experimental data indicating the criterion was not relevant for Pa 215 metabolism (supplementary material S13).

Amino acids with high cellular energy yields (mol ATP (mol amino acid)^−1^) also had high biomass yields (Cmol biomass (mol amino acid)^−1^); the two yields correlated with an r^2^ value of 0.99 (supplementary data S18). Therefore, maximizing rates for cellular energy production or biomass production had similar trends and neither predicted rCCR phenotype (supplementary material S13).

### Single dimension optimization of resource investment predicted overflow metabolism, inconsistent with rCCR phenotypes

Computational analysis was used to identify phenotypes that minimized resource investment into catabolic pathways. Explicit investment models were not possible in non-model organism, *P. aeruginosa*. Therefore, two previously developed resource investment proxies were applied to estimate relative, proteome investment into central metabolism^19,22,49,50^. The flux minimization proxy assumes the total network flux is proportional to the enzymatic resources required to synthesize the necessary proteome^19,22,42,51^. Another proxy for protein investment minimizes the number of enzyme catalyzed reactions (*a.k.a.* minimal proteome investment) identifying the smallest proteome required to realize an in silico phenotype^19^. These hypotheses assumed central metabolism enzymes could be approximated as having the same molecular weight with the same amino acid distribution.

Both proxies for resource investment, when applied as the single optimization criterion, predicted overflow metabolisms for most amino acids (supplementary material S13,S15,S16). The in vitro experimental cultures did not demonstrate substantial overflow metabolisms indicating these single dimension criteria were not relevant for Pa 215 phenotypes.

### Substrate utilization order was consistent with a resource utilization strategy optimizing substrate oxidation and proteome investment

Life occurs in multifactorial environments with multiple stressors influencing phenotypes^20,42,52^. Two dimensional, optimizations of in silico phenotypes were performed where the first dimension considered the optimal conversion of substrate into cellular energy which completely oxidized the substrate. The second dimension approximated the nutrient investment into the enzymes required to realize the in silico phenotype, for example the amount of anabolic nitrogen required to synthesize the proteome or the amount of ATP required to form the associated peptide bonds^19,22^ (supplementary material S15,S16). Both the flux minimization and minimal proteome investment proxies were tested. Two-dimensional optimization (2-DO) using the flux minimization proxy had poor correlations with the observed amino acid utilization order (Fig. 5d). Alternatively, 2-DO using complete substrate oxidation and the minimal proteome proxy predicted the experimental utilization order for amino acids (r^2^ = 0.67) (Fig. 5e).

2-DO was further refined using experimentally measured proteomics data. The constitutively expressed TCA cycle, anaplerotic enzymes, ATP synthase, and electron transport chain (Fig. 3) were considered part of a core, constitutive proteome, independent of substrate. The refined, 2-DO theory considered only the resource investment extraneous to the conserved, core proteome. This theory lead to improved predictions of amino acid utilization order with the minimal proteome investment theory but not the flux minimization theory (Fig. 5d, e). The outlier amino acid in Fig. 5e was serine. Serine is catabolized via the L-serine dehydratase enzyme which is O_2_-labile suggesting higher cell densities and lower O_2_ concentrations were necessary for its functionality^53^. The predictive accuracy of the analysis improved to a correlation of r^2^ = 0.88 if serine data were excluded.

**Figure 6:**
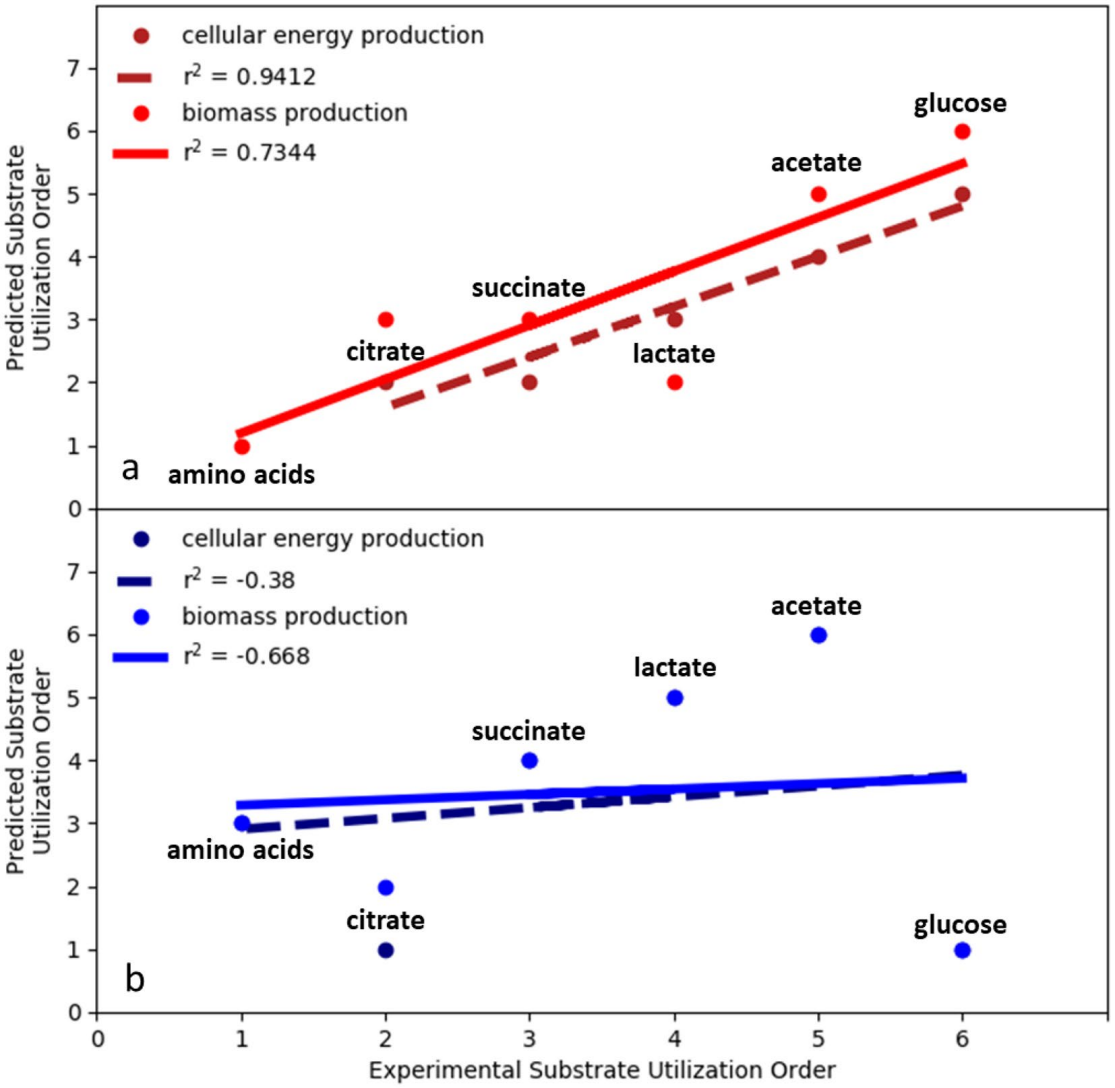
Predicted substrate utilization order, based on in silico analysis, compared to experimental substrate utilization order for cultures of *P. aeruginosa* 215 growing on chemically-defined media. The experimental substrate utilization order was: (1) aggregate pool of amino acids, (2) citrate, (2) succinate, (3) lactate, (4) acetate, and (5) glucose. (**a**) Substrate utilization order predictions for cellular energy production and biomass production using a two-dimensional optimization including complete substrate oxidation and minimal proteome investment. Simulations used a refined, core proteome theory where only enzyme-catalyzed reactions extraneous to the experimentally-measured, constitutive, core proteome where considered. A subset of the core enzymes is shown in Fig. 3 while the explicit list can be found in supplementary material S15. (**b**) Predicted substrate preference based on the ‘maximization of rate’ criterion for cellular energy production and biomass production. In silico product yield on substrate was assumed proportional to rate.

2-DO, considering complete oxidation of substrate and minimal proteome investment, was extended to the other CSP media substrates including organic acids and glucose. Analysis applied the minimal proteome investment with conserved core proteome assumption and considered both cellular energy production as well as the more complex biomass production (Fig. 6a). The experimental substrate utilization hierarchy, which was determined using culturing data from the five CSP media formulations, was used to assess the accuracy of the predictions (Fig. 2). The in silico analysis accurately predicted, substrate utilization order with r^2^ correlations of 0.94 and 0.73 for cellular energy and biomass production, respectively. The cellular energy simulations had a noteworthy correlation with experimental data suggesting ATP production was a superior in silico optimization criterion for Pa 215. The biomass simulations considered an aggregate amino acid substrate pool containing all 20 metabolites, which was not considered for cellular energy simulations. As anticipated, the aggregate amino acid pool greatly reduced the requirement for enzymatic steps by negating de novo amino acid synthesis reactions (Fig. 6a, supplementary material S15,S16). The correlation between the predicted and experimental substrate utilization order for the biomass simulations was not as strong due largely to the predicted order of lactate and citrate utilization. The discrepancy could be due to a couple factors. First, the computational approach approximated the resource investment necessary to synthesize the in silico proteome by assuming all enzyme-catalyzed reactions required the same amount of anabolic resource. This was a necessary simplification due to the lack of detailed data for *P. aeruginosa* that could be improved as more data becomes available. Additionally, the experimental utilization order was based on five separate media formulations which resulted in five separate, dynamic, batch growth profiles each with their own intricacies including cometabolism of substrates.

The maximization of rate criterion was also tested with the additional substrates. The analysis assumed optimal product yields on substrate were proportional to the optimal product rates^43^. The maximum rate criterion did not predict the experimental utilization order for organic acids over glucose. In fact, the predicted utilization order had negative correlations with the experimental data (Fig. 6b). Additional optimizations and aggregate substrate simulations were considered (supplementary material S17–S19). None outperformed the presented approach in terms of accuracy and simplicity.

## Discussion

**Figure 7:**
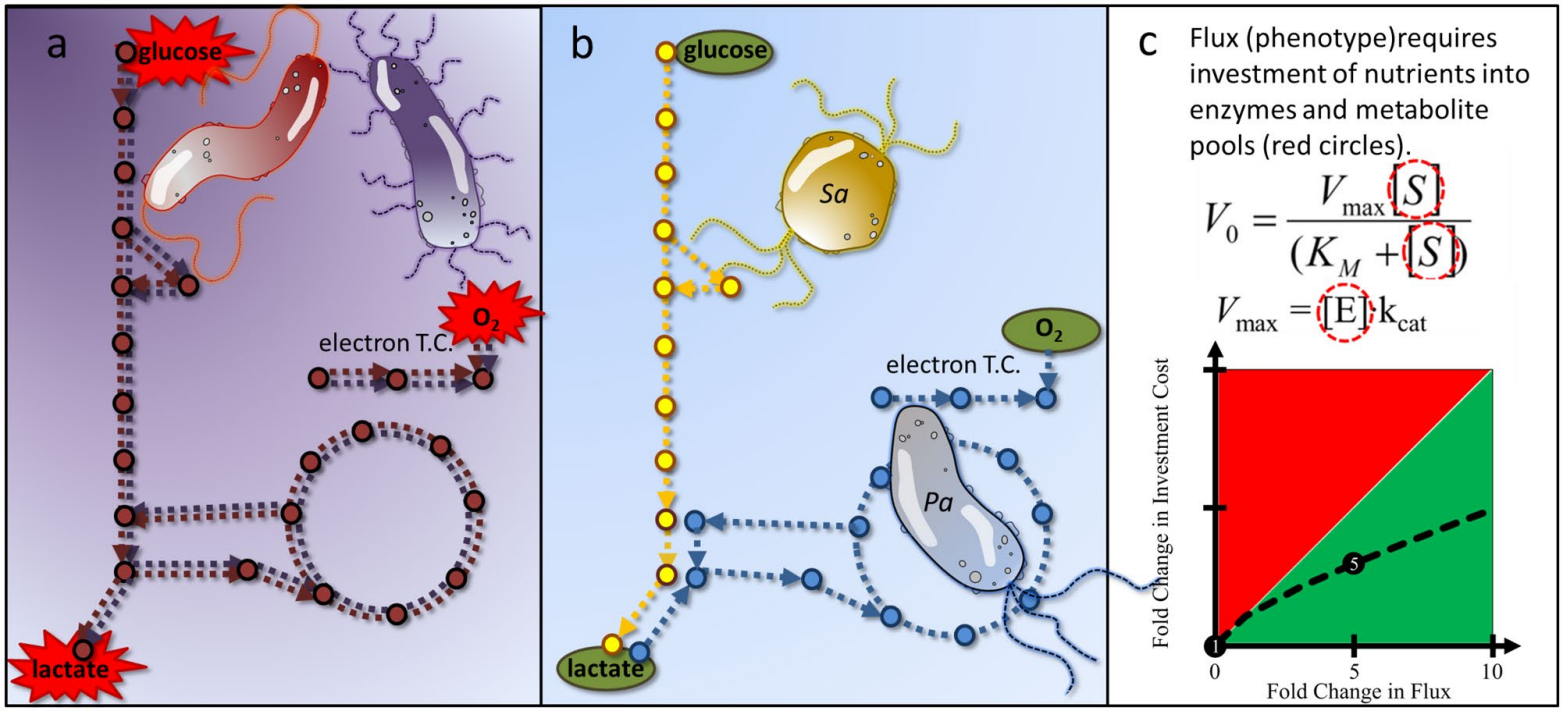
Theoretical consortial interactions including competition and cross feeding. (**a**) Consortium with multiple populations competing for the same electron donor and same electron acceptor and both populations utilizing an overflow metabolism producing inhibitor lactic acid. (**b**) Consortium of rCCR utilizing *P. aeruginosa* (Pa) and cCCR utilizing *S. aureus* (Sa) where substrate preferences are partitioned between the two populations and lactic acid is cross fed. (**c**) Cross feeding can lead to enhanced phenotypic properties, such as flux, for a scarce nutrient pool like reduced carbon or nitrogen. Michaelis–Menten-like kinetics and the requirement to invest resources into both enzyme and metabolite pools leads to a nonlinear relationship between invested resources and enzyme flux. Higher fluxes require a smaller, relative resource investment. See Beck et al*.*^67^ for parameter details.

*P. aeruginosa* preferentially consumes nonfermentable, lower energy substrates, such as succinate over glucose in a strategy known as reverse diauxie or rCCR. The term has been defined in terms of substrate preference relative to cCCR organisms *E. coli* and *B. subtilis*. The rCCR preference for nonfermentable substrates is associated with minimal overflow metabolism and, under certain conditions, can result in cultures preferentially catabolizing substrates that do not maximize cellular growth rates^10,36,54–57^. The CSP media studied here did not result in this property. The term ‘inverse diauxie’ has been proposed to describe microorganisms that prefer substrates that sustain lower growth rates^58^. The rCCR strategy has enabled the broad, global distribution of *P. aeruginosa* in both environmental and medical niches including chronic, diabetic ulcers. The hierarchy of substrate preferences for Pa 215 was: amino acids such as aspartate, followed by citrate, succinate, lactate, acetate, and finally glucose. These preferences were also observed with *P. aeruginosa* PAOI grown on CSP GLAS medium (supplementary material S11). Pa 215 maintained, constitutively, core TCA cycle enzymes and regulated the abundance of the proteins required for specific substrates as needed to convert the substrates into central metabolism intermediates. Analysis using an in silico metabolic model and FBA determined the rCCR phenotype was consistent with a multidimensional, resource utilization strategy where substrate utilization order was based on minimizing the proteome investment required to mineralize the metabolite. Optimization of multiple cellular functions simultaneously has been reported previously for cCCR model organism *E. coli,* albeit with optimization criteria^22,52^. The rCCR phenotypes were not consistent with the commonly applied, systems biology criterion, which maximizes growth rate^43,44^. Pseudomonads are commonly found in consortia and the rCCR metabolism is proposed to provide fitness advantages in these competitive environments^59,60^. Consortia with populations expressing rCCR and cCCR phenotypes have the metabolic basis for an effective division of labor, thus avoiding overlapping substrate preferences which can result in species competition and exclusion^61–65^ (Fig. 7). cCCR organisms prefer primary substrates like glucose where fast growth rate is likely a strong fitness determinant. Glucose can be catabolized by cCCR microorganisms via respiration, fermentation, or a combination of the two strategies. This flexibility enables tradeoffs between high yields during a fully respiratory catabolism and fast rates, with the associated overflow metabolism, during (partially) fermentative catabolism^30,48^. rCCR organisms avoid competition for fermentable carbon sources by preferring to catabolize secondary, byproducts of cCCR metabolism. Fast growth may not be as central to fitness as the efficient extraction of energy from the lower energy substrates. Many rCCR preferred organic acids are nonfermentable which precludes the rCCR metabolism from utilizing the tradeoffs inherent to either high yield or high rate strategies, as commonly observed in cCCR organisms^48^.

Both rCCR and cCCR phenotypes are found in microorganisms described as generalists, e.g*. P. aeruginosa* and *E. coli*. Both rCCR and cCCR phenotypes can be predicted using resource investment theories, albeit with the rCCR organism expressing a respiration-centric phenotype and the cCCR organism expressing a glycolysis-centric, overflow phenotype. Natural environments are often limited by anabolic nutrients including nitrogen^18^. Division of labor, thru rCCR and cCCR based phenotypes, can theoretically enable higher consortia fluxes, from a scarce nitrogen supply, based on the nonlinear relationship between enzyme flux and resource investment^17,66^ (Fig. 7c). This kinetic effect can translate into consortia having a better metabolic return on limiting nutrients, leading to higher biomass accumulation and higher host bioburden^17,67–70^. Additionally, the CCR-based, division of labor could create a positive feedback mechanism by removing inhibitory organic acids and preventing environmental acidification via both organic acid consumption and amino acid catabolism, ultimately increasing consortia productivity by permitting a more complete depletion of substrates^71^. Amino acid catabolism and the release of ammonia can also function as an intercellular communication strategy where the small metabolite influences phenotype, like antibiotic susceptibility, in distant populations^72–74^.

Most virulence mechanisms are nutrient acquisition strategies that are also effective in medical niches^8^. CCR regulates a wide range of social behaviors and likely modulates division of labor which would facilitate substrate acquisition by rCCR microorganisms^17^. *P. aeruginosa* preference for non-fermentable substrates like succinate makes it a secondary resource specialist that requires terminal electron acceptors like O_2_ or nitrate^75^. However, O_2_ is often limiting in biofilms where cellular O_2_ consumption rates are faster than diffusion rates^34,76^. *P. aeruginosa* possesses effective mechanisms to acquire scarce resources like O_2_^70,77–80^. For example, *P. aeruginosa* secretes a cocktail of moieties such as pyocyanin, quinolones, and cyanide^59,81–84^. Exposure to this cocktail can manipulate the *S. aureus* cCCR phenotype, driving it toward overflow and fermentative metabolisms^70,85^. Collectively, the compounds enable a secondary consumer to influence the metabolism of neighboring cells directing their phenotypes toward secreting preferred substrates including organic acids while reserving the O_2_ for *P. aeruginosa*^69,70,84^.

Lactate has remarkable connections to *P. aeruginosa* substrate preference and medical niches including diabetic wounds. Elevated lactate levels found in diabetic wounds come from two sources. First, diabetic patients can have higher levels of serum lactate due to diabetic ketoacidosis, and secondly, lactate is associated with wound bed colonization by bacteria which produce it as a byproduct^86–88^. > 80% of chronic wounds are colonized by *P. aeruginosa*^89^ while 90% of chronic leg ulcers are colonized by *S. aureus*^78,90–93^ which displays a cCCR phenotype^94,95^. Not surprisingly, these bacteria are often co-isolated^55,85,96^. Wounds colonized by multispecies can be more difficult to treat and can have more negative outcomes than wounds colonized by a single species^69,91,93^. Mutualistic interactions in consortia, based on complementary rCCR and cCCR metabolisms, could lead to emergent properties such as enhanced biomass productivity based on enhanced resource acquisition and better metabolic return on investment of scarce nutrients, ultimately leading to greater virulence. Mitigating these consortia, through rational countermeasures, will require quantitative knowledge of the metabolic organization which forms the bases of all virulence mechanisms.

## Materials and methods

### Bacterial strain and cultivation

All experiments used *P. aeruginosa* str. 215, a clinical isolate obtained from a chronic wound^33,97^. Frozen stocks of *P. aeruginosa* 215 were prepared by growing cultures in 10 mL of 1/10 strength tryptone soy broth (TSB) at 37 °C with shaking (150 rpm), mixed with 3 mL of 20% glycerol, and stored at − 80 °C.

Frozen stocks were plated on tryptic soy agar (TSA) at 37 °C for 12 h, five colonies were picked to inoculate 10 mL of *Clostridium, Staphylococcus, Pseudomonas* (CSP) medium in culture tubes. CSP is a chemically defined medium developed to support the growth of *P. aeruginosa, Staphylococcus aureus,* and *Clostridium perfringens* as monocultures or consortia^34^ (supplementary material S1). For CSP supplemented with one or more organic acids, 22 mM of each organic acid specified was added.

A total of three culturing tubes containing 10 mL of CSP were each inoculated with about five colonies from the TSA plates, incubated at 37 °C with shaking at 150 rpm (tubes were placed at a 45° angle in the shaker to increase mixing) and grown until the cultures reach an OD_600_ of 0.5. 1 mL of each culture was then added to 49 mL of fresh CSP medium in 250 mL baffled flasks and an OD_600_ of 0.010. The baffled flasks were capped with gas permeable foam lids and incubated at 37 °C with shaking at 150 rpm. Sampling occurred about every hour during the first 12 h and less frequently afterwards.

### Culture sampling

Samples were drawn from each flask for OD_600_, pH, amino acid, and carbon metabolite measurements. An aliquot of 1.5 mL of culture was collected at each sampling, cells were separated from the supernatant using centrifugation at 7000 rpm for 10 min (Eppendorf 5415D microcentrifuge). Supernatants were then filtered using 0.22 µm syringe filters prior to being stored at − 20 °C.

At each sampling, a volume of culture was collected for OD_600_ measurement. OD_600_ readings were blanked with fresh CSP and samples were diluted, if necessary, to keep OD_600_ measurements ≤ 0.30.

### Organic acid and sugar analyses

HPLC analysis of select carbon metabolites including glucose and organic acids was performed with an Agilent 1200 series HPLC equipped with a refractive index detector (RID) and an Aminex HPX-87H ion exclusion column, 300 mm × 7.8 mm. A mobile phase of 5 mM H_2_SO_4_ was run at a flow rate of 0.6 mL/min for 25 min/injection. A volume of 200 µL of sample was added to an HPLC vial with 200 µL of an internal standard of 1 g/L fucose dissolved in 10 mM H_2_SO_4_. Each sample was injected twice for a total of two technical replicates for each of the three biological replicates for each time point. HPLC analysis of culture supernatants were compared to NMR analysis (Chenomx library and an internal standard of DSS) as a verification of metabolite identities and to ensure no major metabolites were being missed^34^. Supplementary material S3 includes a comparison of HPLC- and NMR-based analyses of select metabolites during batch growth.

### Amino acid analysis

HPLC analysis of amino acids was performed with an Agilent 1100 series equipped with a diode array detector (DAD) and a ZORBAX Eclipse XDB-C18 column, 4.6 mm ID × 250 mm (5 µm) 80 Å. This setup was used with the Agilent protocol for HPLC analysis of amino acids^98^.

### Cell dry weight measurement

A correlation curve between OD_600_ and grams of cell dry weight (g CDW) per liter was constructed. 5 mL aliquots of *P. aeruginosa* culture harvested in mid-exponential growth phase diluted to a range of densities were dried at 80 °C for 24 h in aluminum drying pans and weighed. Correlation equation was: (g CDW/L) = (OD_600_)*2.23^−1^.

### Proteomics analyses

*P. aeruginosa* cultures (n = 3) were collected via centrifugation at 3600×*g* and washed three times with phosphate buffered solution to remove residual media. Cells were resuspended in 1.25 mL radioimmunoprecipitation assay buffer consisting of 12.5 μL of protease inhibitor (Halt Protease Cocktail Inhibitor, Thermo Fisher Scientific, Rockford, IL) to prevent enzymatic degradation upon cell lysis, 0.1 mg/ml lysozyme to solubilize the cell peptidoglycan layer, and 5 mM dithiothreitol (DTT) to cleave protein disulfide bonds. Cells were lysed mechanically in a beadbeater (Mini-beadbeater-1, Biospec Products, Inc, Bartlesville, OK) at 4800 oscillations/min with the remainder of the vial filled with 0.1 mm diameter zirconia/silica beads for a total time of 2.5 min (five cycles at a duration of 30 s each with chilling in an ice water bath between cycles).

Protein concentrations were determined by protein assay kit (DC Bradford Reagent, Thermo Fisher Scientific). 15 µg of proteins were taken from all samples and transferred to centrifugal filter units (Microcon-30 kDa Centrifugal Filter Unit with Ultracel-30 membrane, Millipore Sigma, Billerica, MA). The samples were then processed following the filter aided sample preparation method^99^, in which proteins were reduced with DTT, alkylated with iodoacetamide to prevent disulfide bond reformation, then enzymatically digested overnight into peptides with trypsin at 1:50 enzyme:substrate (w:w) at 37 °C. The resulting peptides were desalted using a C18 column (Macro SpinColumn, Harvard Apparatus, Holliston, MA), dried in a centrifugal evaporator, then resuspended in 5% acetonitrile with 0.1% formic acid to a concentration of 0.2 μg/μL.

200 ng samples of peptides were separated in a HPLC system (1260 Infinity LC System, Agilent, Santa Clara, CA) and a C18 column (3.5 μm particle size, 150 mm length × 75 μm internal diam, Zorbax 300SB, Agilent) using a 60 min mobile phase gradient ranging from 5 to 85% organic (0.1% formic acid in water to 0.1% formic acid in acetonitrile) at 250 nL/min flow rate for a total run time of 75 min. Following LC separation, peptides were ionized by nanoelectrospray with a spray voltage of 1.90 kV and 275 °C capillary temperature, then analyzed in a high resolution Orbitrap mass spectrometer (Orbitrap Velos Pro purchased in 2007, Thermo Scientific, Waltham, MA) with automatic gain control set at 10^6^ ions and injection times of 1–200 ms. Full scan mass spectra from m/z 400 to 2000 at 30,000 mass resolution were collected in data-dependent acquisition mode. Ten precursor ions were selected from each full mass scan for analysis by tandem mass spectrometry (MS/MS, using 30% energy in HCD mode for fragmentation).

Raw mass spectra data files were processed for protein identification using the MaxQuant software (v. 1.5.3.30)^100^ with main search parameters of 4.5 ppm peptide tolerance, 20 ppm MS/MS match tolerance, 10 ppm MS/MS de novo tolerance, seven minimum peptide length, carbamidomethyl as fixed modification, 0.01 FDR, oxidation and acetylation variable modification, and enabled search for contaminants. Protein abundances were further data processed and normalized with log transformation (base 2) for data visualization and statistical analyses (ANOVA, *p* value < 0.05) using the Perseus software (v.1.5.4.0)^101^. The Search Tool for Retrieval of Interacting Genes (STRING) database (v. 10.5)^102^ was used for protein–protein interactions amongst the statistically significant proteins, set at medium confidence of 0.4 and protein annotation (functional enrichment analyses).

### In silico analysis of metabolism and resource allocation

A genome-scale, stoichiometric model of *P. aeruginosa* (iMO1086)^40,41^ was analyzed using flux balance analysis (FBA) via the COBRA Toolbox (https://opencobra.github.io/cobratoolbox/stable/cite.html) in MATLAB using the Gurobi optimization program (http://www.gurobi.com) (supplementary material S12,S20). Carbon and O_2_ limitations were modeled by setting the carbon (5 mmol/g/h) or O_2_ (20 mmol/g/h) uptake rates, respectively, for each of the examined carbon sources and maximizing the production of biomass or cellular energy (i.e. quantified as the number of ATP bonds hydrolyzed). Enzyme limitation was modeled by minimizing the number of participating reactions for specified substrate uptake rates while producing biomass or cellular energy. Suboptimal solutions between the minimal total flux and the maximum product yield (biomass or cellular energy) or the minimum proteome and the maximum product yield (biomass or cellular energy) were identified by minimizing an aggregate objective function. The aggregate objective function was the sum of either total flux or total proteome and a weighted flux through the substrate transport reaction of interest. Optimization between proteome investment and product yield was achieved by changing the weight of the flux through the carbon or oxygen transport reaction of interest. The algorithms can be found in supplementary material S20.

## Supplementary Information

Supplementary Tables.

## References

[1] Byrd MS (2011). Direct evaluation of *Pseudomonas aeruginosa* biofilm mediators in a chronic infection model. Infect. Immun..

[2] Behrends V (2013). Metabolic adaptations of *Pseudomonas aeruginosa* during cystic fibrosis chronic lung infections. Environ. Microbiol..

[3] Calhoun JH, Murray CK, Manring MM (2008). Multidrug-resistant organisms in military wounds from Iraq and Afghanistan. Clin. Orthop. Relat. Res..

[4] Frykberg RG, Banks J (2015). Challenges in the treatment of chronic wounds. Adv. Wound Care New Rochelle.

[5] Jarbrink K (2017). The humanistic and economic burden of chronic wounds: A protocol for a systematic review. Syst. Rev..

[6] Fife CE, Carter MJ (2012). Wound care outcomes and associated cost among patients treated in US outpatient wound centers: Data from the US wound registry. Wounds.

[7] Valot B (2015). What it takes to be a *Pseudomonas aeruginosa*? The core genome of the opportunistic pathogen updated. PLoS ONE.

[8] Rojo F (2010). Carbon catabolite repression in Pseudomonas: Optimizing metabolic versatility and interactions with the environment. FEMS Microbiol. Rev..

[9] Görke B, Stülke J (2008). Carbon catabolite repression in bacteria: Many ways to make the most out of nutrients. Nat. Rev. Microbiol..

[10] Collier DN, Hager PW, Phibbs PV (1996). Catabolite repression control in the Pseudomonads. Res. Microbiol..

[11] *Scitable by Nature EDUCATION* 2005).

[12] Pellett S, Bigley DV, Grimes DJ (1983). Distribution of *Pseudomonas aeruginosa* in a riverine ecosystem. Appl. Environ. Microb..

[13] Döring G (1996). Distribution and transmission of *Pseudomonas aeruginosa* andBurkholderia cepacia in a hospital ward. Pediatr. Pulmonol..

[14] Romling U, Kader A, Sriramulu DD, Simm R, Kronvall G (2005). Worldwide distribution of *Pseudomonas aeruginosa* clone C strains in the aquatic environment and cystic fibrosis patients. Environ. Microbiol..

[15] Hamilton WA, Dawes E, A. (1959). A diauxic effect with *Pseudomonas aeruginosa*. Biochem. J..

[16] Liu Y, Gokhale CS, Rainey PB, Zhang XX (2017). Unravelling the complexity and redundancy of carbon catabolic repression in Pseudomonas fluorescens SBW25. Mol. Microbiol..

[17] Park H, McGill SL, Arnold AD, Carlson RP (2019). Pseudomonad reverse carbon catabolite repression, interspecies metabolite exchange, and consortial division of labor. Cell.Mol. Life Sci..

[18] Sterner RW, Elser JJ (2002). Ecological Stoichiometry: The Biology of Elements from Molecules to the Biosphere.

[19] Carlson RP (2007). Metabolic systems cost-benefit analysis for interpreting network structure and regulation. Bioinformatics.

[20] Carlson, R. P., Oshota, O. J. & Taffs, R. L. in *Reprogramming Microbial Metabolic Pathways* (eds Xiaoyuan Wang, Jian Chen, & Peter Quinn) 139–157 (Springer, Netherlands, 2012).

[21] Folsom JP, Carlson RP (2015). Physiological, biomass elemental composition and proteomic analyses of *Escherichia coli* ammonium-limited chemostat growth, and comparison with iron- and glucose-limited chemostat growth. Microbiology.

[22] Carlson RP (2009). Decomposition of complex microbial behaviors into resource-based stress responses. Bioinformatics.

[23] Goelzer A, Fromion V (2011). Bacterial growth rate reflects a bottleneck in resource allocation. Biochim. Biophys. Acta.

[24] Goelzer A, Fromion V (2017). Resource allocation in living organisms. Biochem. Soc. Trans..

[25] Yang L (2016). solveME: Fast and reliable solution of nonlinear ME models. BMC Bioinform..

[26] Beg QK (2007). Intracellular crowding defines the mode and sequence of substrate uptake by *Escherichia coli* and constrains its metabolic activity. Proc. Natl. Acad. Sci. USA.

[27] Vazquez A, Oltvai ZN (2016). Macromolecular crowding explains overflow metabolism in cells. Sci. Rep..

[28] Zhuang K, Vemuri GN, Mahadevan R (2011). Economics of membrane occupancy and respiro-fermentation. Mol. Syst. Biol..

[29] Szenk M, Dill KA, de Graff AMR (2017). Why do fast-growing bacteria enter overflow metabolism? Testing the membrane real estate hypothesis. Cell Syst..

[30] Basan M (2015). Overflow metabolism in *Escherichia coli* results from efficient proteome allocation. Nature.

[31] Folsom JP, Parker AE, Carlson RP (2014). Physiological and proteomic analysis of *Escherichia coli* iron-limited chemostat growth. J. Bacteriol..

[32] Schuster S, Boley D, Moller P, Stark H, Kaleta C (2015). Mathematical models for explaining the Warburg effect: A review focussed on ATP and biomass production. Biochem. Soc. Trans..

[33] Woods J (2012). Development and application of a polymicrobial in vitro wound biofilm model. J. Appl. Microbiol..

[34] Yung YP (2019). Reverse diauxie phenotype in *Pseudomonas aeruginosa* biofilm revealed by exometabolomics and label-free proteomics. NPJ Biofilms Microbiomes.

[35] Behrends V, Ebbels TM, Williams HD, Bundy JG (2009). Time-resolved metabolic footprinting for nonlinear modeling of bacterial substrate utilization. Appl. Environ. Microbiol..

[36] Berger A (2014). Robustness and plasticity of metabolic pathway flux among uropathogenic isolates of *Pseudomonas aeruginosa*. PLoS ONE.

[37] Nouwens AS (2000). Complementing genomics with proteomics: The membrane subproteome of*Pseudomonas aeruginosa* PAO1. Electrophoresis.

[38] Penesyan A (2015). Genetically and phenotypically distinct *Pseudomonas aeruginosa* cystic fibrosis isolates share a core proteomic signature. PLoS ONE.

[39] Nikel PI, Chavarria M, Fuhrer T, Sauer U, de Lorenzo V (2015). Pseudomonas putida KT2440 strain metabolizes glucose through a cycle formed by enzymes of the Entner-Doudoroff, Embden-Meyerhof-Parnas, and pentose phosphate pathways. J. Biol. Chem..

[40] Phalak P, Chen J, Carlson RP, Henson MA (2016). Metabolic modeling of a chronic wound biofilm consortium predicts spatial partitioning of bacterial species. BMC Syst. Biol..

[41] Oberhardt MA, Goldberg JB, Hogardt M, Papin JA (2010). Metabolic network analysis of *Pseudomonas aeruginosa* during chronic cystic fibrosis lung infection. J. Bacteriol..

[42] Schuetz R, Kuepfer L, Sauer U (2007). Systematic evaluation of objective functions for predicting intracellular fluxes in *Escherichia coli*. Mol. Syst. Biol..

[43] Schuster S, Pfeiffer T, Fell DA (2008). Is maximization of molar yield in metabolic networks favoured by evolution?. J. Theor. Biol..

[44] Varma A, Boesch BW, Palsson BO (1993). Stoichiometric interpretation of *Escherichia coli* glucose catabolism under various oxygenation rates. Appl. Environ. Microbiol..

[45] Varma A, Palsson BO (1994). Stoichiometric flux balance models quantitatively predict growth and metabolic by-product secretion in wild-type *Escherichia coli* W3110. Appl. Environ. Microb..

[46] Bar-Even A (2011). The moderately efficient enzyme: Evolutionary and physicochemical trends shaping enzyme parameters. Biochemistry.

[47] Volkmer B, Heinemann M (2011). Condition-dependent cell volume and concentration of *Escherichia coli* to facilitate data conversion for systems biology modeling. PLoS ONE.

[48] Novak M, Pfeiffer T, Lenski RE, Sauer U, Bonhoeffer S (2006). Experimental tests for an evolutionary trade-off between growth rate and yield in *E. coli*. Am. Nat..

[49] Hoffmann S, Hoppe A, Holzhütter H-G (2006). Composition of metabolic flux distributions by functionally interpretable minimal flux modes (MinModes). Genome Inf..

[50] Holzhutter HG (2004). The principle of flux minimization and its application to estimate stationary fluxes in metabolic networks. Eur. J. Biochem..

[51] Carlson RP, Taffs RL (2010). Molecular-level tradeoffs and metabolic adaptation to simultaneous stressors. Curr. Opin. Biotechnol..

[52] Schuetz R, Zamboni N, Zampieri M, Heinemann M, Sauer U (2012). Multidimensional optimality of microbial metabolism. Science New York NY.

[53] Velayudhan J, Jones MA, Barrow PA, Kelly DJ (2004). l-Serine catabolism via an oxygen-labile l-serine dehydratase is essential for colonization of the avian gut by *Campylobacter jejuni*. Infect. Immun..

[54] Frimmersdorf E, Horatzek S, Pelnikevich A, Wiehlmann L, Schomburg D (2010). How *Pseudomonas aeruginosa* adapts to various environments: a metabolomic approach. Environ. Microbiol..

[55] Tiwari N, Campbell J (1969). Enzymatic control of the metabolic activity of Pseudomonas aeruginosa grown in glucose or succinate media. Biochimica et Biophysica Acta BBA Gen. Subj..

[56] Trautwein K (2012). Benzoate mediates repression of C(4)-dicarboxylate utilization in "Aromatoleum aromaticum" EbN1. J. Bacteriol..

[57] Kremling A, Geiselmann J, Ropers D, de Jong H (2018). An ensemble of mathematical models showing diauxic growth behaviour. BMC Syst. Biol..

[58] Kremling A, Geiselmann J, Ropers D, de Jong H (2015). Understanding carbon catabolite repression in *Escherichia coli* using quantitative models. Trends Microbiol..

[59] Ibberson CB, Whiteley M (2020). The social life of microbes in chronic infection. Curr. Opin. Microbiol..

[60] King AN, de Mets F, Brinsmade SR (2020). Who's in control? Regulation of metabolism and pathogenesis in space and time. Curr. Opin. Microbiol..

[61] Tuncil YE (2017). Reciprocal prioritization to dietary glycans by gut bacteria in a competitive environment promotes stable coexistence. MBio.

[62] Goyal A, Dubinkina V, Maslov S (2018). Multiple stable states in microbial communities explained by the stable marriage problem. ISME J..

[63] Ren D, Madsen JS, Sorensen SJ, Burmolle M (2015). High prevalence of biofilm synergy among bacterial soil isolates in cocultures indicates bacterial interspecific cooperation. ISME J..

[64] Russel J, Roder HL, Madsen JS, Burmolle M, Sorensen SJ (2017). Antagonism correlates with metabolic similarity in diverse bacteria. Proc. Natl. Acad. Sci. USA.

[65] Brileya KA, Camilleri LB, Zane GM, Wall JD, Fields MW (2014). Biofilm growth mode promotes maximum carrying capacity and community stability during product inhibition syntrophy. Front. Microbiol..

[66] Carlson RP (2018). Competitive resource allocation to metabolic pathways contributes to overflow metabolisms and emergent properties in cross-feeding microbial consortia. Biochem. Soc. Trans..

[67] Beck, A., Hunt, K., Bernstein, H. C. & Carlson, R. in *Biotechnology for Biofuel Production and Optimization* (eds Carrie A. Eckert & Cong T. Trinh) 407–432 (Elsevier, Amsterdam, 2016).

[68] Hillesland KL, Stahl DA (2010). Rapid evolution of stability and productivity at the origin of a microbial mutualism. Proc. Natl. Acad. Sci. USA.

[69] DeLeon S (2014). Synergistic interactions of *Pseudomonas aeruginosa* and Staphylococcus aureus in an in vitro wound model. Infect. Immun..

[70] Filkins LM (2015). Coculture of *Staphylococcus aureus* with *Pseudomonas aeruginosa* drives *S. aureus* towards fermentative metabolism and reduced viability in a cystic fibrosis model. J. Bacteriol..

[71] Bernstein HC, Paulson SD, Carlson RP (2012). Synthetic *Escherichia coli* consortia engineered for syntrophy demonstrate enhanced biomass productivity. J. Biotechnol..

[72] Bernier SP, Letoffe S, Delepierre M, Ghigo JM (2011). Biogenic ammonia modifies antibiotic resistance at a distance in physically separated bacteria. Mol. Microbiol..

[73] Palkova Z (1997). Ammonia mediates communication between yeast colonies. Nature.

[74] Wang J, Yan D, Dixon R, Wang YP (2016). Deciphering the principles of bacterial nitrogen dietary preferences: A strategy for nutrient containment. mBio.

[75] Schreiber K (2007). The anaerobic regulatory network required for *Pseudomonas aeruginosa* nitrate respiration. J. Bacteriol..

[76] Stewart PS (2003). Diffusion in biofilms. J. Bacteriol..

[77] Cornforth DM, Foster KR (2013). Competition sensing: The social side of bacterial stress responses. Nat. Rev. Microbiol..

[78] Korgaonkar A, Trivedi U, Rumbaugh KP, Whiteley M (2013). Community surveillance enhances *Pseudomonas aeruginosa* virulence during polymicrobial infection. Proc. Natl. Acad. Sci. USA.

[79] Wang M, Schaefer AL, Dandekar AA, Greenberg EP (2015). Quorum sensing and policing of *Pseudomonas aeruginosa* social cheaters. Proc. Natl. Acad. Sci. USA.

[80] Allegretta G (2017). In-depth profiling of MvfR-regulated small molecules in *Pseudomonas aeruginosa* after quorum sensing inhibitor treatment. Front. Microbiol..

[81] Deziel E (2004). Analysis of *Pseudomonas aeruginosa* 4-hydroxy-2-alkylquinolines (HAQs) reveals a role for 4-hydroxy-2-heptylquinoline in cell-to-cell communication. Proc. Natl. Acad. Sci. USA.

[82] Meirelles LA, Newman DK (2018). Both toxic and beneficial effects of pyocyanin contribute to the lifecycle of *Pseudomonas aeruginosa*. Mol. Microbiol..

[83] Hall S (2016). Cellular effects of pyocyanin, a secreted virulence factor of *Pseudomonas aeruginosa*. Toxins Basel.

[84] Price-Whelan A, Dietrich LE, Newman DK (2006). Rethinking 'secondary' metabolism: Physiological roles for phenazine antibiotics. Nat. Chem. Biol..

[85] Noto MJ, Burns WJ, Beavers WN, Skaar EP (2017). Mechanisms of pyocyanin toxicity and genetic determinants of resistance in *Staphylococcus aureus*. J. Bacteriol..

[86] James TJ, Hughes MA, Cherry GW, Taylor RP (2000). Simple biochemical markers to assess chronic wounds. Wound Repair. Regen..

[87] Trengove NJ, Langton SR, Stacey MC (1996). Biochemical analysis of wound fluid from nonhealing and healing chronic leg ulcers. Wound Repair. Regen..

[88] Cox K (2012). Prevalence and significance of lactic acidosis in diabetic ketoacidosis. J. Crit. Care.

[89] de Oliveira FP (2017). Prevalence, antimicrobial susceptibility, and clonal diversity of *Pseudomonas aeruginosa* in Chronic Wounds. J. Wound Ostomy Contin. Nurs..

[90] Rhoads DD, Wolcott RD, Sun Y, Dowd SE (2012). Comparison of culture and molecular identification of bacteria in chronic wounds. Int. J. Mol. Sci..

[91] Dalton T (2011). An in vivo polymicrobial biofilm wound infection model to study interspecies interactions. PLoS ONE.

[92] Kirketerp-Moller K (2008). Distribution, organization, and ecology of bacteria in chronic wounds. J. Clin. Microbiol..

[93] Murray JL, Connell JL, Stacy A, Turner KH, Whiteley M (2014). Mechanisms of synergy in polymicrobial infections. J. Microbiol..

[94] Ferreira MT, Manso AS, Gaspar P, Pinho MG, Neves AR (2013). Effect of oxygen on glucose metabolism: Utilization of lactate in *Staphylococcus aureus* as revealed by in vivo NMR studies. PLoS ONE.

[95] Tynecka Z, Szcześniak Z, Malm A, Los R (1999). Energy conservation in aerobically grown *Staphylococcus aureus*. Res. Microbiol..

[96] Sanchez CJ (2013). Biofilm formation by clinical isolates and the implications in chronic infections. BMC Infect. Dis..

[97] James GA (2008). Biofilms in chronic wounds. Wound Repair. Regen..

[98] Bacon CW, White J (2000). Microbial Endophytes.

[99] Mann, M. *Filter Aided Sample Preparation (FASP) Method*. http://www.biochem.mpg.de/226356/FASP (2013).

[100] Tyanova S, Temu T, Cox J (2016). The MaxQuant computational platform for mass spectrometry-based shotgun proteomics. Nat. Protocols.

[101] Tyanova S (2016). The Perseus computational platform for comprehensive analysis of (prote)omics data. Nat. Methods.

[102] Szklarczyk D (2015). STRING v10: Protein–protein interaction networks, integrated over the tree of life. Nucleic Acids Res..

